# Health economic evaluation of stereotactic body radiotherapy (SBRT) for hepatocellular carcinoma: a systematic review

**DOI:** 10.1186/s12962-019-0198-z

**Published:** 2020-01-10

**Authors:** Haiyin Wang, Chunlin Jin, Liang Fang, Hui Sun, Wendi Cheng, Shanlian HU

**Affiliations:** 1 Shanghai Health Development Research Center (Shanghai Medical Information Center), 1477 Beijing Road (West), Shanghai, China; 2 Public Health School, Fudan University, Shanghai, China

**Keywords:** Stereotactic body radiotherapy, Hepatocellular carcinoma, Cost-effectiveness, Systemic review

## Abstract

Stereotactic body radiotherapy (SBRT) is a novel noninvasive treatment for hepatocellular carcinoma. SBRT can achieve effective local control, but it requires a relatively high input of resources; this systematic review was performed to assess the cost effectiveness of SBRT in the treatment of hepatocellular carcinoma to provide a basis for government pricing and medical insurance decision-making. The PubMed, EMBASE, Cochrane Library, CNKI, Wanfang and SinoMed databases were searched to collect economic evaluations of SBRT for the treatment of hepatocellular carcinoma from the date of database inception through December 31, 2018. Two reviewers independently screened the studies, extracted the data and performed a descriptive analysis of the basic characteristics, methods of economic evaluation and main results, as well as the quality and heterogeneity of the reports. A total of 5 studies were included. Among them, the level of heterogeneity was relatively acceptable, with a median score of 90%. Four studies were cost-utility analyses (CUAs), and 1 was a cost-effectiveness analysis (CEA). The incremental cost effectiveness ratio (ICER) for sorafenib compared to SBRT was US $114,795 per quality-adjusted life year gained (cost/QALY) in patients with advanced hepatocellular carcinoma. The ICER for proton beam therapy compared to SBRT was US $6465 in patients with inoperable advanced hepatocellular carcinoma. The ICER for SBRT compared to RFA was US $164,660 for patients with unresectable colorectal cancer (CRC) with liver metastases and US $56,301 for patients with early-stage hepatocellular carcinoma. For patients with inoperable localized hepatocellular carcinoma, compared with RFA–SBRT therapy, the ICERs for SBRT–SBRT and SBRT–RFA were US $558,679 and US $2197,000, respectively; RFA–RFA was dominated. In conclusion, there is limited evidence suggesting that SBRT could be cost-effective for highly specific subpopulations of HCC patients, and further economic evaluations based on randomized controlled trials (RCTs) or cohort studies are needed.

## Background

Recent studies have shown that the incidence and mortality of primary liver cancer rank 7th and 2nd, respectively, in 185 countries [1]. Hepatocellular carcinoma (HCC) cases account for the majority of primary liver cancer cases (75–85%). The incidence of and mortality due to primary liver cancer in China are high and rank 4th and 2nd, respectively, among cancers [2]. The disability-adjusted life years (DALYs) specific to primary liver cancer in the Chinese population in 2016 were 1153.9 per 10,000 person-years, accounting for 54.6% of the global DALYs. The age-standardized DALY rate specific to primary liver cancer was 844.1 per 100,000 and was three times higher than the average level worldwide [3]. In China, the cost of medical treatment for HCC is increasing [4], which imposes a heavy economic burden.

The treatment measures for and survival rates of HCC vary based on the stage. Early-stage HCC [Barcelona Clinic Liver Cancer (BCLC) stage O/I] can be treated by surgical resection, radiofrequency ablation (RFA), transcatheter arterial chemoembolization (TACE), or liver transplantation. However, the majority of HCC patients are already in the advanced stage when the disease is diagnosed. Currently, there is no treatment that can significantly improve the survival rate of patients with advanced HCC [5]. Surgical resection is an acceptable treatment method for noncirrhotic patients; the cure rate is the highest, and the 5-year survival rate is 41–74% [6]. RFA is the standard treatment for patients with BCLC stage O/I disease who are not candidates for surgery [7]. RFA is suitable for patients with localized HCC, and the 5-year overall survival (OS) rate is 33–55% [8]. For patients with unresectable HCC or TACE contraindications, sorafenib is the standard treatment, and the median survival time is 2.8 months [9].

Stereotactic body radiotherapy (SBRT) is a novel noninvasive technology. It can accurately target tumors with high-dose conformal radiation [10, 11]. Many studies have indicated that SBRT achieves effective local control with acceptable toxicity; in addition, the local control rate within 1–3 years ranges from 80 to 99% [12–15].

However, because SBRT consumes more resources in terms of planning and design, real-time motion management, and 3D multimodal image acquisition, the cost associated with SBRT is high. Many provinces and cities in China and other countries have not included SBRT in the list of chargeable items; therefore, the application of SBRT faces many challenges. Because public hospital cost control measures and medical insurance payment reform have been further strengthened in China and the rest of the world [16], cost effectiveness analyses are being used when developing charge lists and making medical insurance decisions. In a systematic evaluation, this study integrated evidence from existing health economic assessments of the treatment of HCC with SBRT to provide a basis for the promotion of medical insurance payments and charges for SBRT in China and other counties.

## Methods

### Inclusion and exclusion criteria

Research type: This study was a health economic assessment; the types of studies included were not limited (cost-effectiveness analyses (CEAs), cost-utility analyses (CUAs), and cost-benefit analyses (CBAs) were all eligible).

Target population: The target population was clinically diagnosed HCC patients; the specific types of HCC were not limited and included early-stage disease, advanced-stage disease, and metastatic disease.

Interventional measure: SBRT was adopted.

Outcome indicator: The incremental cost-effectiveness ratio (ICER) was adopted.

The exclusion criteria were as follows: ① duplicate studies; ② unoriginal studies, such as reviews and commentaries; ③ studies that only involved SRBT treatment; and ④ studies lacking relevant data that could not be obtained after communicating with the authors.

### Literature search strategy

The PubMed, EMBASE, Cochrane Library [Health Technology Assessment and the National Health Service (NHS) Economic Evaluation Database], China Knowledge Resource Integrated (CNKI), Wanfang, and SinoMed databases were searched for publications of health economic assessments of the use of SBRT to treat HCC. The search period was from the establishment of each database through December 31, 2018. In addition, references in the included studies were investigated to identify additional relevant articles. The study was performed using a combination of Medical Subject Headings (MeSH) and keywords. The English search terms included “stereotactic body radiotherapy”, “SBRT”, “SABR”, “stereotactic ablative radiotherapy”, “EBRT”, “external beam radiotherapy”, “HCC”, “hepatoma”, “hepato* carcinoma”, “cost effectiveness”, “cost utility”, and “cost-benefit”. Using PubMed as an example, the specific search strategy is shown in Additional file 1: Box 1.

### Literature screening, data extraction, quality evaluation of included studies, and normative evaluation of reporting

Two reviewers independently screened the literature, extracted the data, and performed the analyses. Disagreements were submitted to a panel for discussion. A homemade data extraction table was used. The extracted content was as follows: ① the basic information of the included studies, including the first author, publication year, study methods, target population, research perspective, and intervention and control techniques; ② the methods and major results of the health economic assessment, including the parameter setting for the transition probability, cost, effect, discount, and willingness-to-pay threshold; and ③ key elements of the quality evaluation.

Critical appraisal of the included studies was performed using the Consolidated Health Economic Evaluation Reporting Standards (CHEERS) tool, and scores were calculated for all studies: complete reporting was 1 point, partial reporting was 0.5 points, and no reporting and non-applicable reporting was 0 points. Considering the condition that some entries were not applicable, the total number of entries that could be scored was adjusted to calculate the percentage of the actual score to reflect the true satisfaction of the reporting specifications.

## Results

### Literature screening procedure and results

**Fig.1:**
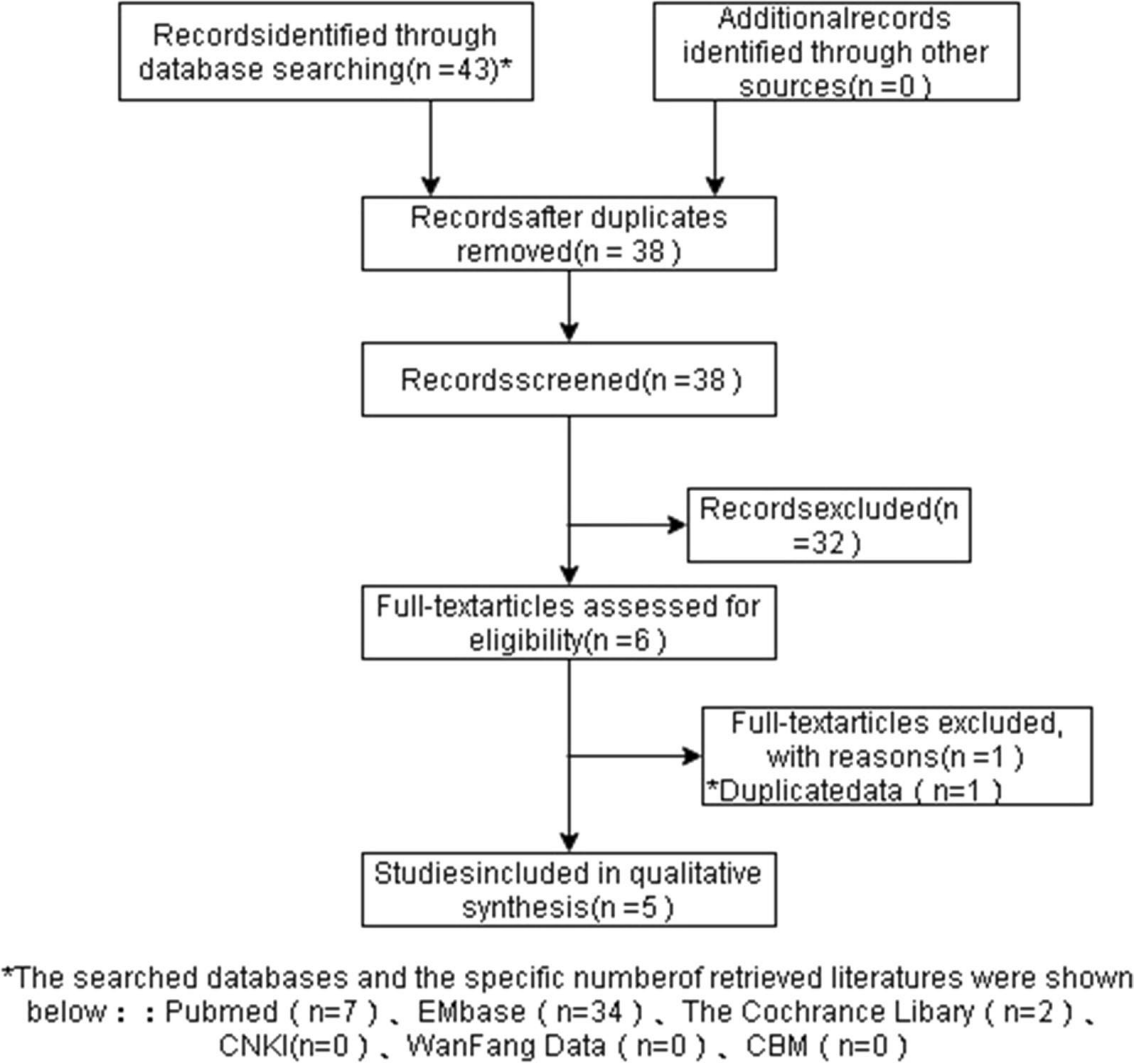
Flow chart of literature inclusion screening

We identified 38 non duplicated papers by searching the aforementioned electronic databases. Of these, 38 potentially relevant articles were screened, and 6 full-text articles met the inclusion criteria. A total of 5 studies were finally included [17–21]. Among them, 4 studies were CUAs, and 1 was a CEA. All the CUAs were based on the Markov model [17–20], and the CEA was a retrospective study based on a database [21]. The literature screening procedure and results are shown in Fig. 1.

### Basic characteristics of included studies and normative evaluation of reporting

#### Basic characteristics of the included studies

The screening process resulted in the inclusion of a total of five studies. Four studies adopted a payer perspective, while one employed a social perspective. Two studies were from Taiwan [18, 20], and three studies were from the USA [17, 19, 21]. Three studies investigated inoperable advanced HCC [18–20], one study investigated early-stage HCC [21], and one study investigated colorectal cancer (CRC) liver metastases [17]. In the control group, three studies used RFA [17, 19, 21], one study used sorafenib [18], and one study used proton beam therapy [20]. In the four Markov model studies [17–20], two simulated a lifetime cycle [17, 19], while two used 5 years [18, 20], and the cycle lengths were generally 1 month. The two studies conducted by Leung et al. both included 3 states [18, 20], and the studies by Kim et al. [17] and Pollom et al. [19] further classified the states and used treatment as a link. The model parameters were mainly obtained from the literature. The parameters of the two models conducted by Leung et al. were mainly obtained from two studies, and the transition probability was calculated. For the threshold value, the two studies from the USA used US $100,000, and the two Taiwanese studies used three times the per capita gross domestic product (GDP) (Tables 1 and 2). In the CEA, survival data were collected from the Surveillance, Epidemiology, and End Results (SEER)-Medicare-linked database (2004–2011), and cost data were compiled using Medicare Part A, B, and D data files. In addition, propensity score adjustment was used.

**Table 1:** Basic characteristics of included studies

Included research	Region	Research type	Study method	Study perspective	Study population	Intervention technique	Control comparator technique	Outcome indicator
Hayeon Kim [17]	USA	CUA^f^	Markov model	Payer	Unresectable CRC liver metastases	SBRT^a^	RFA^b^	ICER^c^
Leung [18]	Taiwan	CUA	Markov model	Payer	Advanced HCC	Sorafenib	SBRT	ICER
Pollom [19]	USA	CUA	Markov model	Social	Inoperable localized HCC	SBRT	RFA	ICER
Leung [20]	Taiwan	CUA	Markov model	Payer	Inoperable advanced HCC	Proton beam therapy	SBRT	ICER, NMBs^c^
Parikh [21]	USA	CEA^g^	Retrospective study	Payer	Early stage HCC	SBRT	RFA	ICER^e^

^a^ Stereotactic body radiotherapy; ^b^ radiofrequency ablation; ^c^ incremental cost effect ratio; ^d^ net monetary benefits; ^e^ life-year gained instead of quality life-year gained. ^f^ cost-utility analysis;^g^ cost-effectiveness analysis

**Table 2:** Design of the SBRT treatment of HCC models in studies

Variable	Hayeon Kim [17]	Leung [18]	Pollom [19]	Leung [20]
Cycle period	Every month	Every month	Every month	
Time horizon	Lifetime	5 years	Lifetime	5 years
Natural history state setting				
Stable disease	No disease progression, stable after treatment	Stable disease	Stable disease after treatment, stable disease after local progression	Stable disease
Progression of disease	Local recurrence, local or distant metastasis	Disease progression	Local metastasis, distant metastasis	Disease progression
Death	Death	Death	Death	Death
Parameter setting and source				
Transition probability	Systemic literature review	Source was one RCT and one sorafenib and SBRT in clinical trial of advanced HCC; the transition probability of the health state was calculated using a formula	Systemic literature review	Source was one stage I/II RCT of SBRT and one stage II RCT
Life quality	Systemic literature review	Source was one RCT and one sorafenib and SBRT in clinical trial of advanced HCC	Systemic literature review	Source was one stage I/II RCT of SBRT and one stage II RCT
Cost	Source was one 2014 Medicare payment dataset of the total treatment cost including the potential complication costs, hospitalization, retreatment, and palliative chemotherapy	Source was from 2015 National Health Insurance Research Database of Taiwan, which was mainly the direct medical cost including drug costs, laboratory tests, physician visits, pharmacy dispensing fees, administration and nursing care fees	Source was 2015 Medicare Services physician fee schedule	Source was from 2016 National Health Insurance Research Database of Taiwan, which was mainly the direct medical cost, including drug costs, laboratory tests, physician visits, pharmacy dispensing fees, and treatment costs for grade 3/4 adverse events
Discount rate	3%	3%	3%	3%
Sensitivity analysis				
Method	One-way and probabilistic sensitivity analyses	One-way and probabilistic sensitivity analyses	One-way and probabilistic sensitivity analyses	One-way and probabilistic sensitivity analyses
Choice of variable	All parameters	All parameters	All parameters	All parameters
Threshold value (each QALY)	$100,000	NT $2213,145	$100,000	NT $2157,024

### Normative evaluation of reporting in included studies

The critical appraisal was performed using the CHEERS tool. The overall reporting normativity of the included studies was high, and the median score was 90%; therefore, the quality was excellent. The reporting normativity of the two model studies (M = 96%) was higher than that of the other three studies. These five studies had poor reporting of the “source of funding” and the discount rate (Table 3).

**Table 3:** Normative evaluation of reporting in the health economic assessment of SBRT in the treatment of HCC (CHEERS scale)

Entry	Hayeon Kim [17]	Leung [18]	Pollom [9]	Leung [20]	Parikh [21]
Title and abstract					
1. Title	Y	Y	Y	Y	Y
2. Abstract	Y	Y	Y	P	Y
Introduction					
3. Background and objective	Y	Y	Y	Y	Y
Methods					
4. Target population and subgroups	P	P	Y	P	Y
5. Setting and location	Y	Y	Y	Y	Y
6. Study perspective	Y	Y	Y	Y	Y
7. Comparators	Y	Y	Y	Y	Y
8. Time horizon	Y	Y	Y	Y	Y
9. Discount rate	P	P	P	P	–
10. Indicators of health outcomes	Y	Y	Y	Y	Y
11. Measurement of effectiveness					
11a Effectiveness estimates^1^	–	–	–	–	Y
11b Effectiveness estimates^1^	P	Y	P	Y	–
12. Measurement and valuation of preference-based outcomes	Y	Y	Y	Y	Y
13. Estimating resources and costs					
13a. Estimating resources and costs	–	–	–	–	Y
13b. Estimating resources and costs	Y	Y	Y	Y	–
14. Currency, price date, and conversion	Y	Y	Y	Y	–
15. Choice of model	Y	Y	Y	Y	–
16. Model assumptions	Y	Y	Y	Y	–
17. Analytical methods	Y	Y	Y	Y	Y
Results					
18. Study parameters	Y	Y	Y	Y	Y
19. Incremental costs and outcomes	Y	Y	Y	Y	Y
Characterizing uncertainty					
20a. Characterizing uncertainty	–	–	–	–	Y
20b. Characterizing uncertainty	Y	Y	Y	Y	–
21. Characterizing heterogeneity	–	–	–	–	–
Discussion					
22. The consistency of major findings, limitations and generalizability of the article with current knowledge	Y	Y	Y	Y	Y
Other					
23. Source of funding	N	Y	Y	N	N
24. Conflicts of interest	Y	Y	Y	Y	Y
Actual score^a^	20.5	22	22	20.5	18
Adjusted total score^b^	23	23	23	23	20
Adjusted score (%)^c^	89	96	96	89	90

*Y* complete reporting (1 point); *P* partial reporting (0.5 points); *N* no reporting (0 point); -: non-applicable reporting (0 points)

^1^ a, b corresponded to population and model studies, respectively (the same hereafter); ^a^ actual evaluation score; ^b^ adjusted total score was the total score of the article after non-applicable entries were excluded; ^c^ adjusted score = actual score/adjusted total score *100%

### Major results of the included studies

Three studies compared SBRT and RFA. The populations in these three studies were different, and the assessment results were also different. For early-stage HCC, SBRT was not cost-effective compared to RFA, and the ICER value was US $56,301, which was lower than the given threshold value (when it is less expensive and less effective, the desired ratio is higher than the threshold). For inoperable localized HCC and unresectable CRC liver metastases, there was no cost-effective treatment. The cost-effectiveness was compared among different treatment combinations of SBRT and RFA for inoperable localized HCC. Using RFA–SBRT as the baseline, the RFA–RFA strategy was the dominated treatment strategy (obviously not cost-effective). The ICER values of the two combinations, SBRT–RFA and SBRT–SBRT, were both higher than the given threshold value and did not exhibit obviously superior cost-effectiveness. One study compared SBRT and sorafenib, the only drug that can treat advanced HCC. The results showed that when SBRT was used as the baseline, sorafenib was not clearly more cost-effective. One study reported a comparison between SBRT and proton beam therapy. Using SBRT as the baseline and 3 times the per capita GDP of Taiwan of that year as the threshold, the ICER value of proton beam therapy was $6465, which was lower than the payment threshold value ($65,364), indicating that it is cost-effective (Table 4).

**Table 4:** Major results of health economic assessment of SBRT in the treatment of HCC

Included research	Intervention technique	Control technique	Incremental cost	Incremental output	ICER	Payment threshold	Basic conclusion
Hayeon Kim [17]	SBRT^c^	RFA^d^	8202	0.050	164,660	100,000^a^	Not cost-effective
Leung [18]	Sorafenib	SBRT	969,041	0.260	3788,238	2213,145^b^	Not cost-effective
Pollom [19]	SBRT–SBRT	RFA-SBRT	4269	0.008	558,679	100,000^a^	Not cost-effective
	SBRT–RFA	RFA–SBRT	4,394	0.002	2197,000	100,000^a^	Not cost-effective
	RFA–RFA	RFA–SBRT	283	− 0.012	–	100,000^a^	Dominated
Leung [20]	Proton beam therapy	SBRT	557,907	2.610	213,354	2157,024^b^	Cost-effective
Parikh [21]	SBRT	RFA	− 1967	− 0.035	56,301	100,000^a^	Not cost-effective

^a^ The cost unit of incremental cost, ICER, and threshold value were in US dollars; ^b^ The cost unit of incremental cost, ICER, and threshold value were in New Taiwan dollars; ^c^ stereotactic body radiotherapy; ^d^ radiofrequency ablation; % ICER per life-year gained, not quality life-year gained

## Discussion

This article systemically evaluated health economic assessments related to HCC treatment with SBRT. The results showed that there are limited numbers of existing studies. Four studies out of the 5 that were found were model studies. Three articles were from the USA, and two articles were from Taiwan. There is currently no health economic assessment evidence from mainland China. Using the threshold value evaluated by the authors, SBRT was cost-effective when compared to sorafenib for the treatment of advanced HCC and was cost-effective when compared to RFA for the treatment of early-stage HCC. In patients with unresectable CRC liver metastases and late-stage HCC, SBRT was not cost-effective compared to RFA and proton beam therapy. In the SBRT and RFA combination treatment regimens, the SBRT–SBRT and SBRT–RFA strategies had an extension strategy but were not cost-effective.

Among the studies included in this assessment, one of the four model studies used the social perspective. The other four studies used the payer perspective to calculate only the direct medical cost and did not consider direct nonmedical costs and indirect costs, which led to underestimations of the cost, resulting in a lower ICER. Considering that SBRT requires few visits and that the patients are mostly elderly individuals, we think that this perspective should not be a significant factor in reimbursement decision-making. One study used the combination technique to assess cost-effectiveness. The results showed that RFA-SBRT was the dominant strategy; the cost increased, but the utility value decreased. Therefore, it is not recommended for use in combination in clinical practice.

This study included four model studies. Two studies used the threshold value of US $100,000, and two studies used the threshold value of 3 times the per capita GDP set by the World Health Organization (WHO), which varies by year. With the development of value-based medicine pricing in recent years, the threshold concept has been further expanded in some countries and regions. For example, the threshold in the UK takes into account the cost and QALY (by weighting), which considers the disease burden, broader social benefits, and treatment innovations and improvements. In addition, threshold values are adjusted for rare diseases and tumors [22, 23]. Therefore, in a comparison of the cost-effectiveness of SBRT and other relevant techniques, the conclusions may be different, leading to uncertainty. If all the studies had used similar willingness-to-pay thresholds, such as $100,000, the ICER for sorafenib compared with SBRT would have been US $114,795, indicating that it is not cost-effective. The ICER for proton beam therapy compared with SBRT would have been US $6465, indicating that it is cost-effective; the conclusion of the original analysis is supported and reliable.

In the two Taiwanese studies, the transition probability and utility value were primarily based on one RCT and one clinical trial, whereas the two US evaluations used parameters from dozens of studies. In the choice of parameters, there were mainly stage I and II clinical trial data; therefore, the results might be different from actual market data. In addition, conversion estimations of the utility value calculated with a formula might cause bias in the results.

Sensitivity analyses were performed in all the included studies. For the treatment of unresectable CRC liver metastases, SBRT was not cost-effective. However, considering that the survival period substantially influenced the stability of the result, the authors proposed that when the survival period of SBRT-treated patients was extended for 1 month or when SBRT was applied in the population of patients with tumors larger than 4 cm, SBRT was cost-effective.

The quality of the evidence still needs to be strengthened; in particular, there were few multicenter RCTs. The choice of parameters from multiple sources involves many factors that may influence the results; for example, population characteristics, disease stages, metastases, and complications might all influence the cost and utility value. Although the results of the sensitivity analyses were all reliable, attention should be given to these aspects in the application of these conclusions.

The current study has some limitations. ① Only the publicly available literature was searched. This might give rise to publication bias. ② The literature screening and quality evaluation processes had subjective factors. ③ The number of included studies was low. Only a qualitative analysis was performed, and quantitative integration was not performed.

## Conclusion

The results of this study suggest that there is limited evidence supporting the cost-effectiveness of SBRT for highly specific subpopulations of HCC patients, and more evidence from RCTs or cohort studies is needed for validation. It is recommended that Chinese researchers perform original studies in this area to provide direct evidence to support payment decisions.

## Supplementary information

**Additional file 1: Box 1. **The PubMed search strategy.

## Data Availability

All data generated or analyzed during this study are included in the published article.
