# Supplementary material for: Health economic evaluation of stereotactic body radiotherapy (SBRT) for hepatocellular carcinoma: a systematic review
Source: Cost Eff Resour Alloc. 2020 Jan 10;18:1. doi: 10.1186/s12962-019-0198-z (PMC6954573; doi:10.1186/s12962-019-0198-z)
Supplement: Supplementary file 1 — Additional file 1: Box 1. The PubMed search strategy. [file 12962_2019_198_MOESM1_ESM.docx]

Additional file 1

Box 1 The PubMed search strategy

| #1 | Search "Liver Neoplasms"[MeSH] |
| --- | --- |
| #2 | Search "Carcinoma, Hepatocellular"[MeSH] |
| #3 | Search "liver cancer" or "hepatoma" or "hepato* carcinoma" |
| #4 | #1 OR #2 OR #3 |
| #5 | Search "Cost-Benefit Analysis"[MeSH] |
| #6 | Search "cost effectiveness" OR "cost utility" OR "cost benefit" |
| #7 | #5 OR #6 |
| #8 | Search "SBRT" OR "SABR" OR "EBRT" OR "stereotactic body radiation therapy" OR "stereotactic ablative radiotherapy" OR "external beam radiotherapy" |
| #9 | #4 AND #7 AND #8 |
